# Supplementary figures and images for: Layers of immunity: Deconstructing the Drosophila effector response
Source: eLife. 2025 Nov 5;14:RP107030. doi: 10.7554/eLife.107030 (PMC12588608; doi:10.7554/eLife.107030)

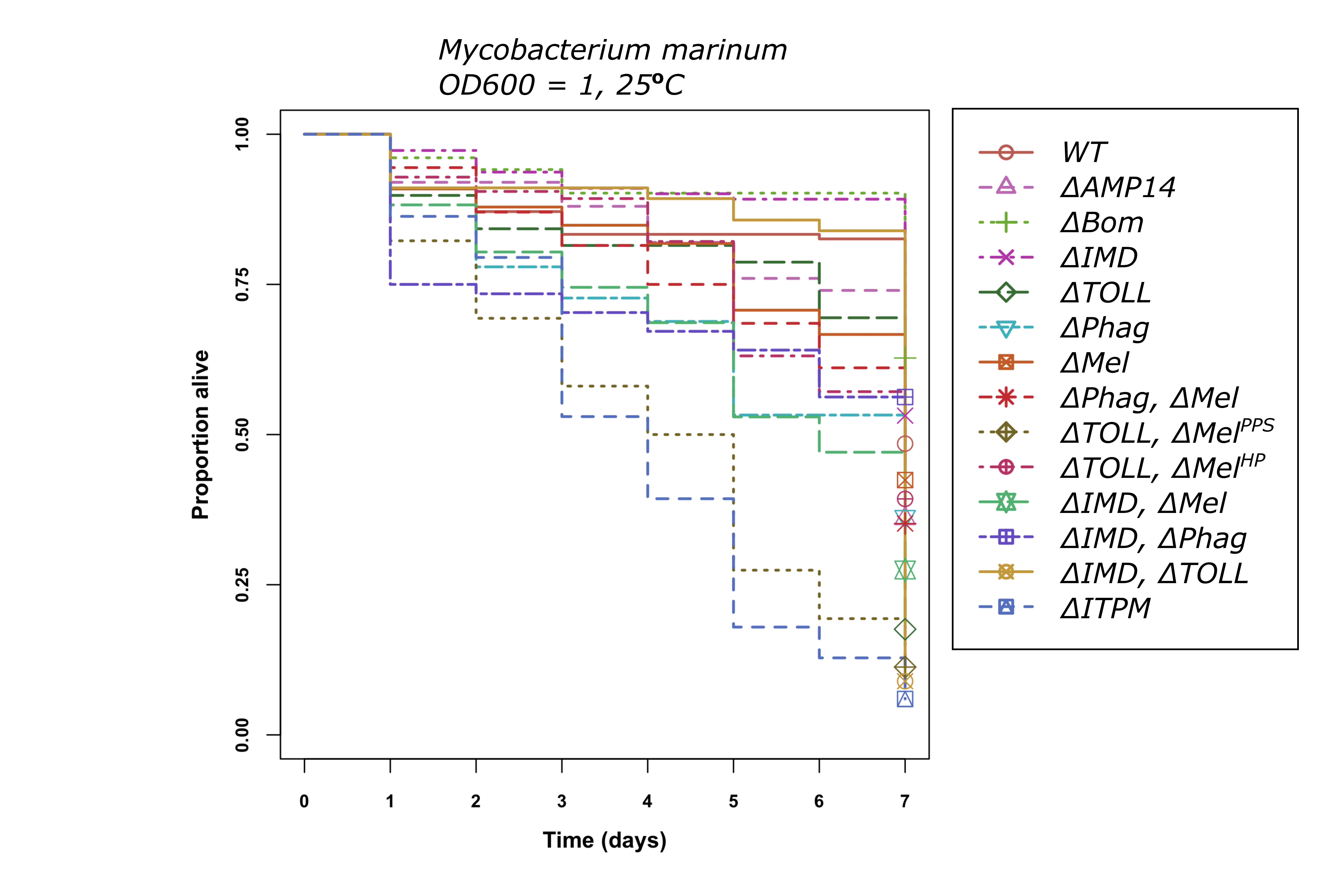

Supplement: Supplementary file 2. [file elife-107030-supp2.zip › Supplementary file 2/Gram-positive/My. marinum.png]

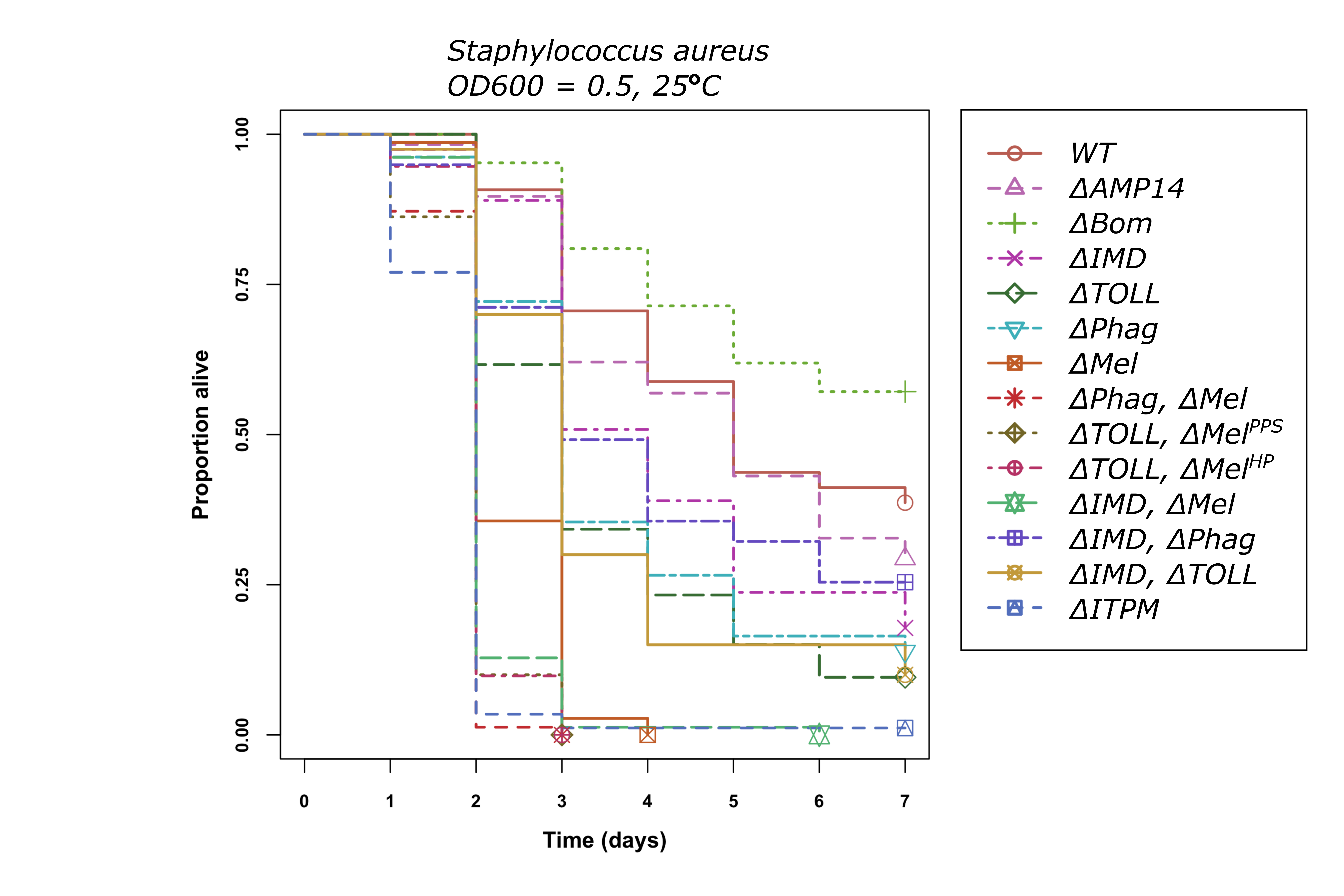

Supplement: Supplementary file 2. [file elife-107030-supp2.zip › Supplementary file 2/Gram-positive/Sta. aureus.png]

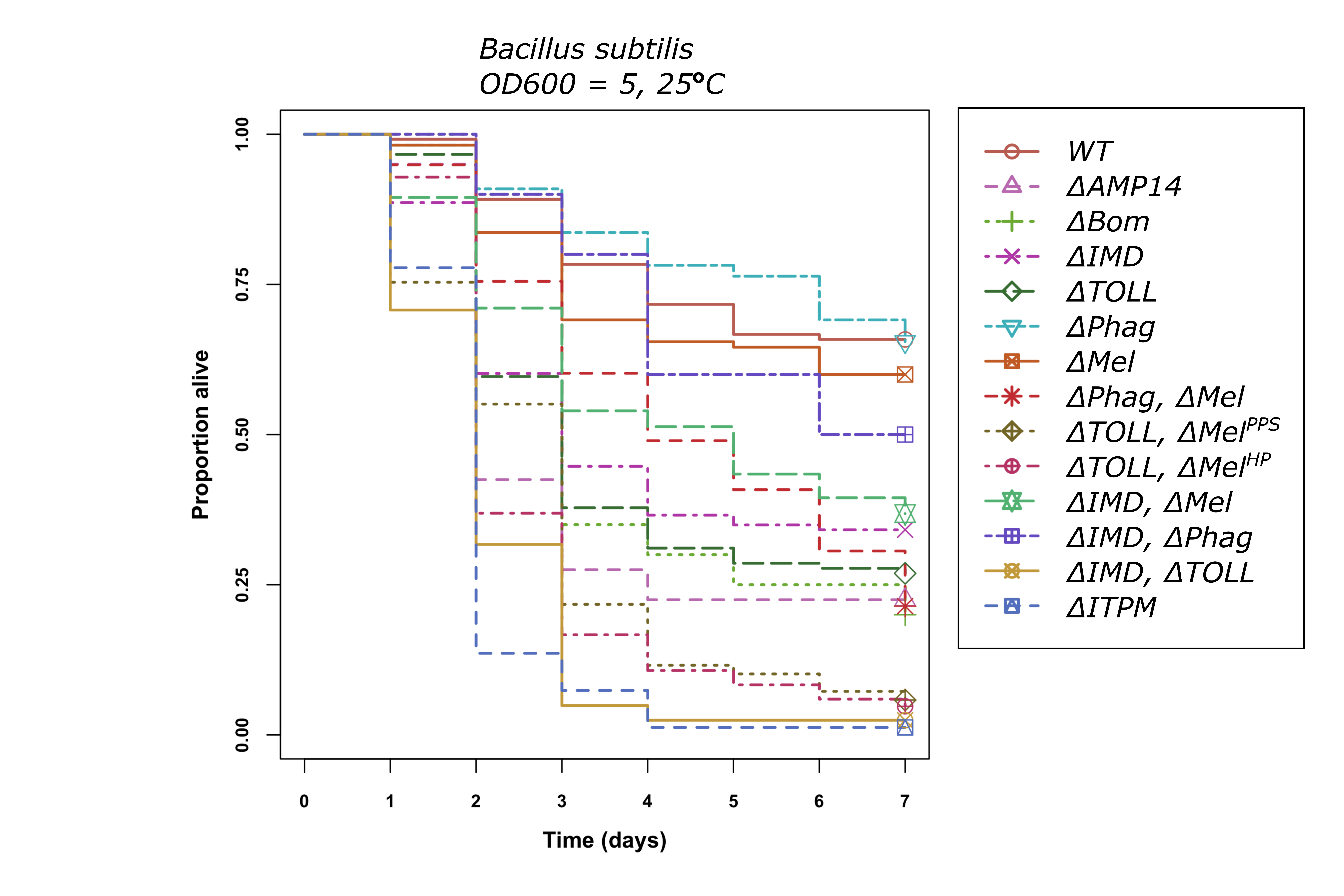

Supplement: Supplementary file 2. [file elife-107030-supp2.zip › Supplementary file 2/Gram-positive/B. subtilis.png]

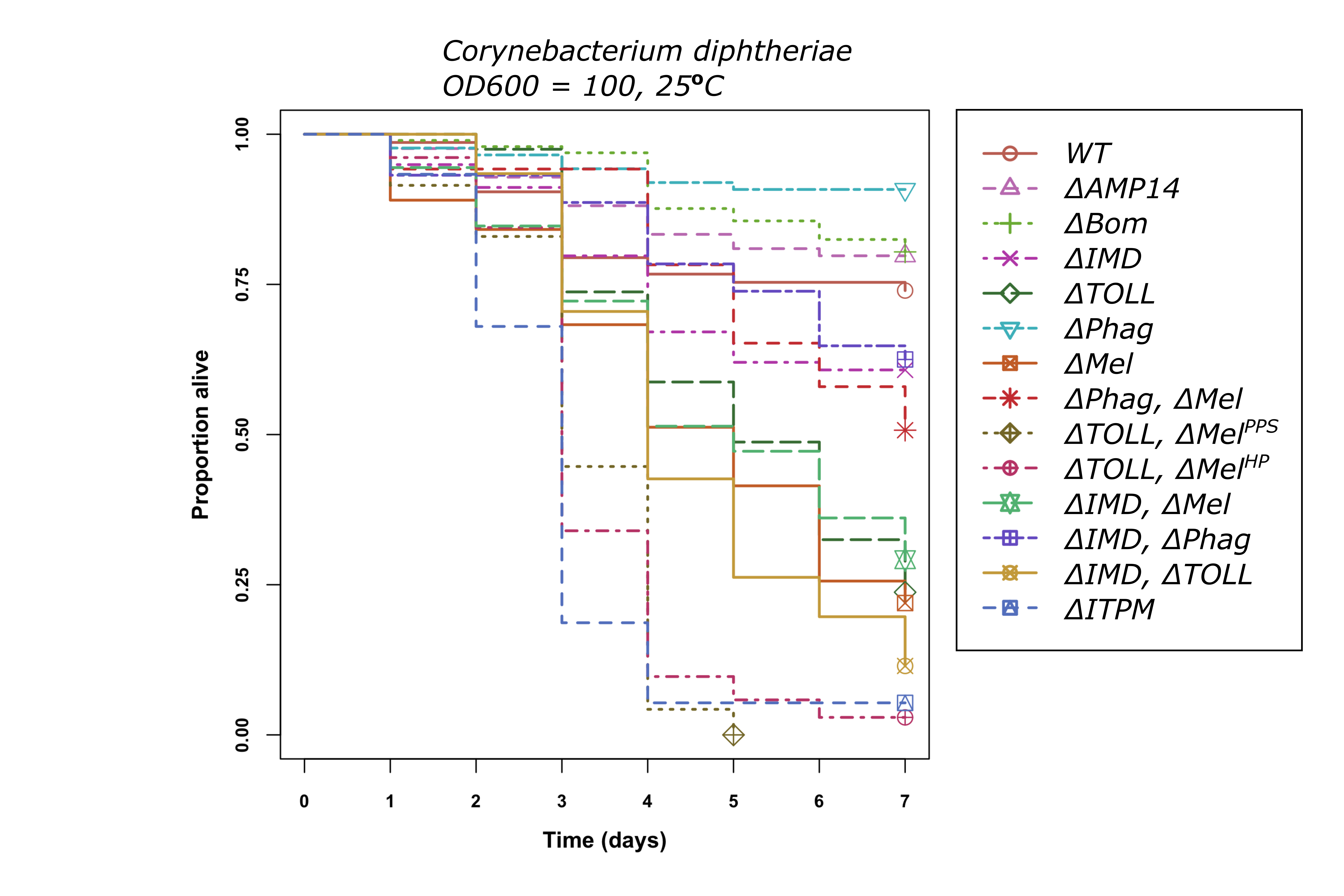

Supplement: Supplementary file 2. [file elife-107030-supp2.zip › Supplementary file 2/Gram-positive/C. diphtheriae.png]

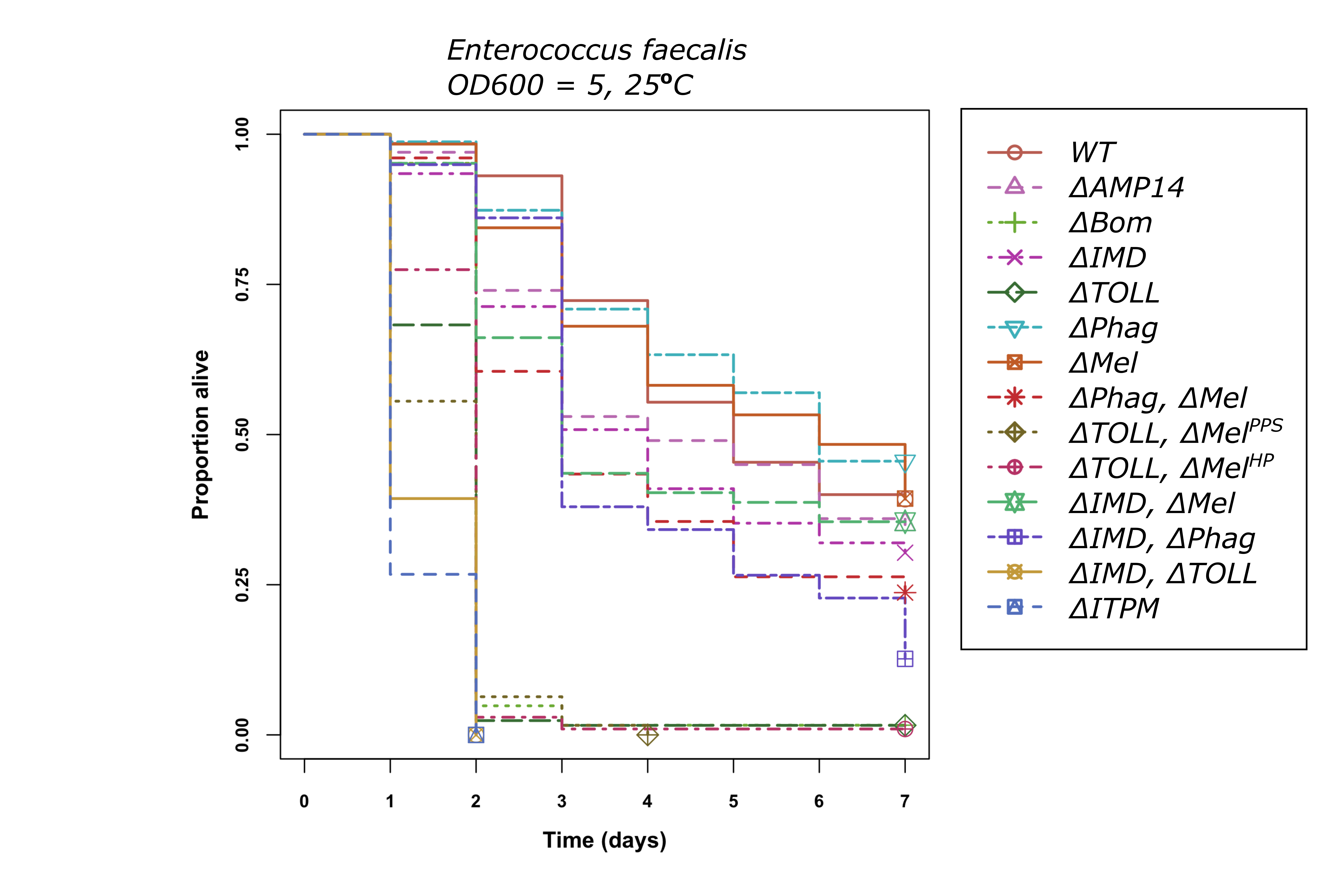

Supplement: Supplementary file 2. [file elife-107030-supp2.zip › Supplementary file 2/Gram-positive/E. faecalis.png]

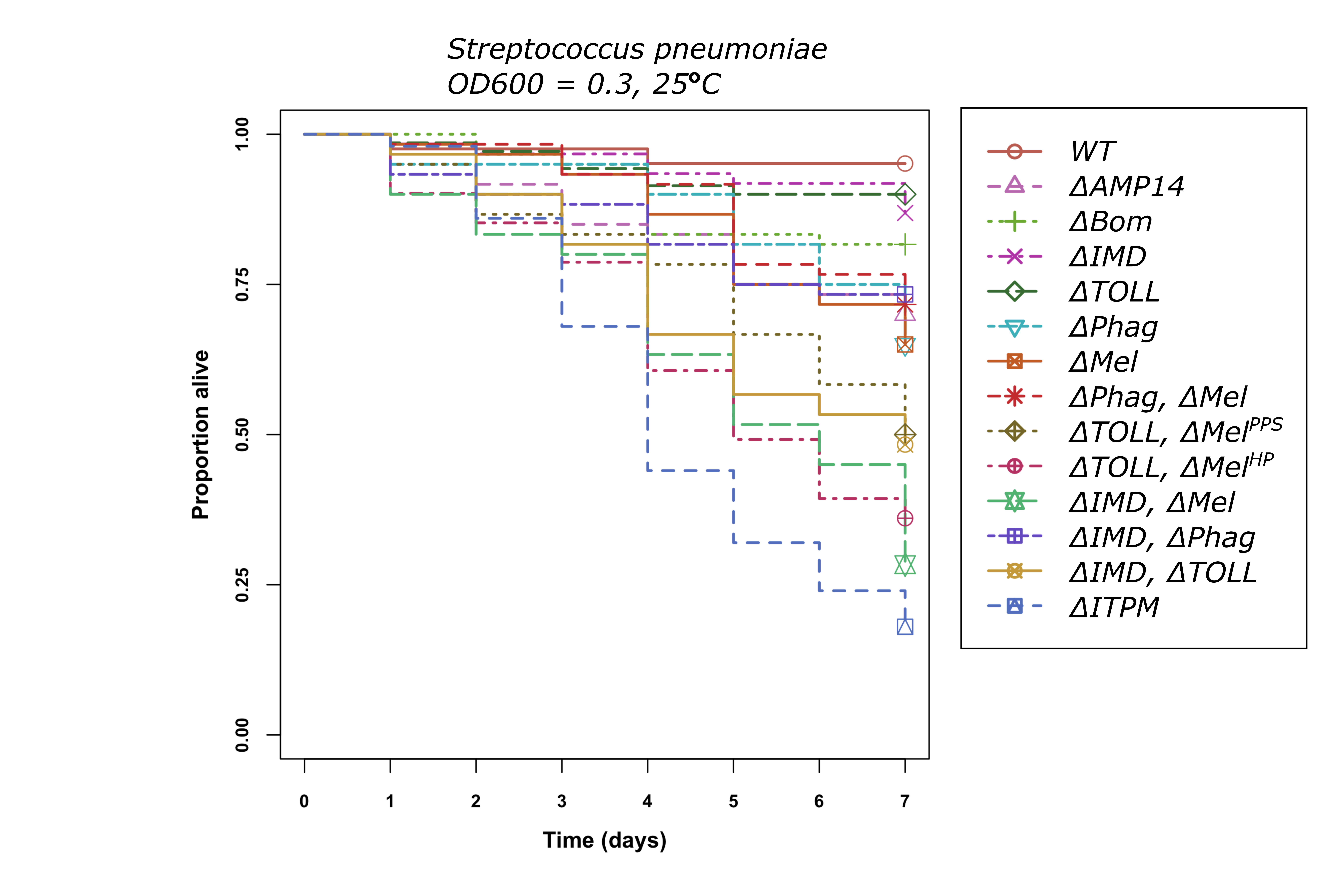

Supplement: Supplementary file 2. [file elife-107030-supp2.zip › Supplementary file 2/Gram-positive/Str. pneumoniae.png]

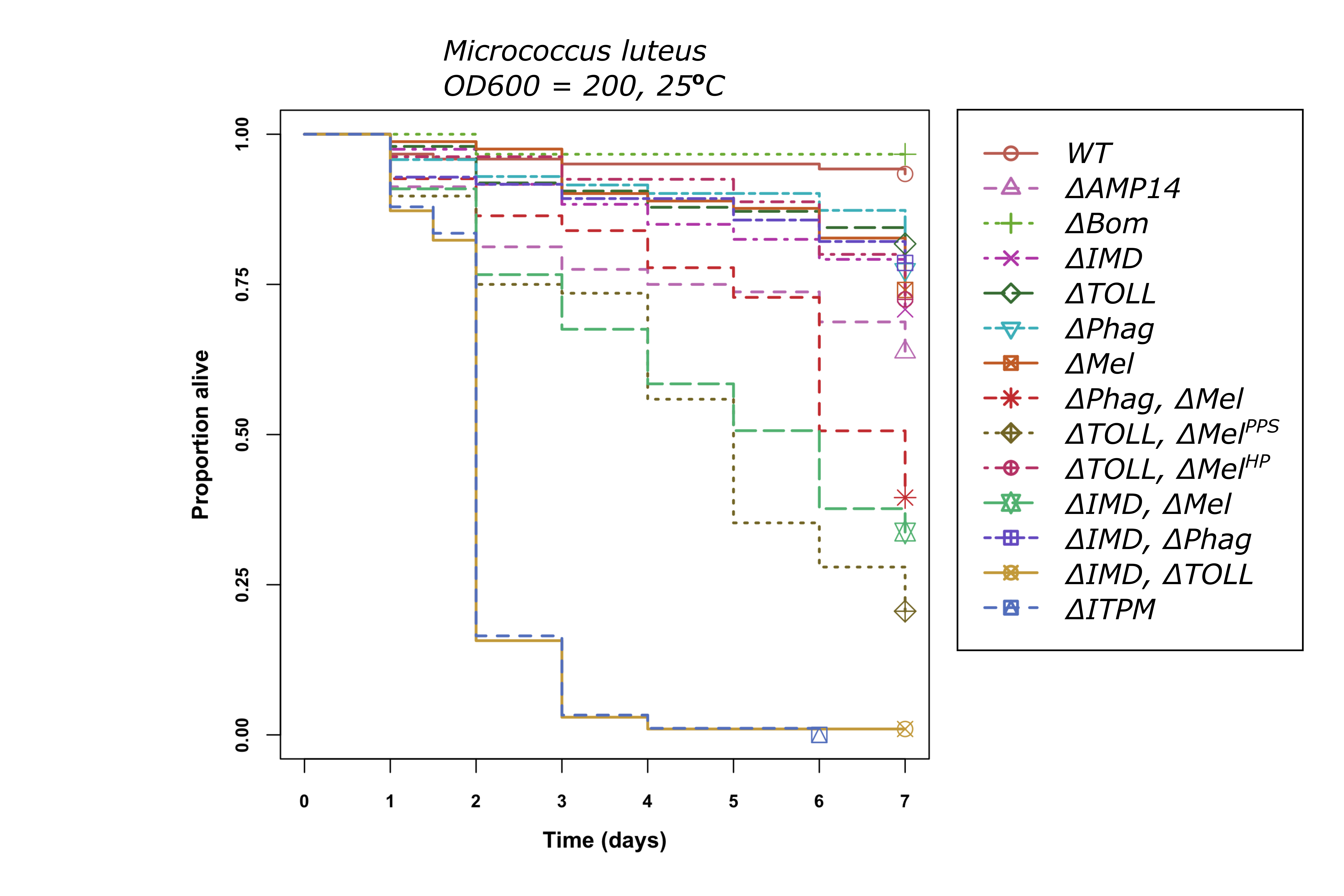

Supplement: Supplementary file 2. [file elife-107030-supp2.zip › Supplementary file 2/Gram-positive/Mi. luteus.png]

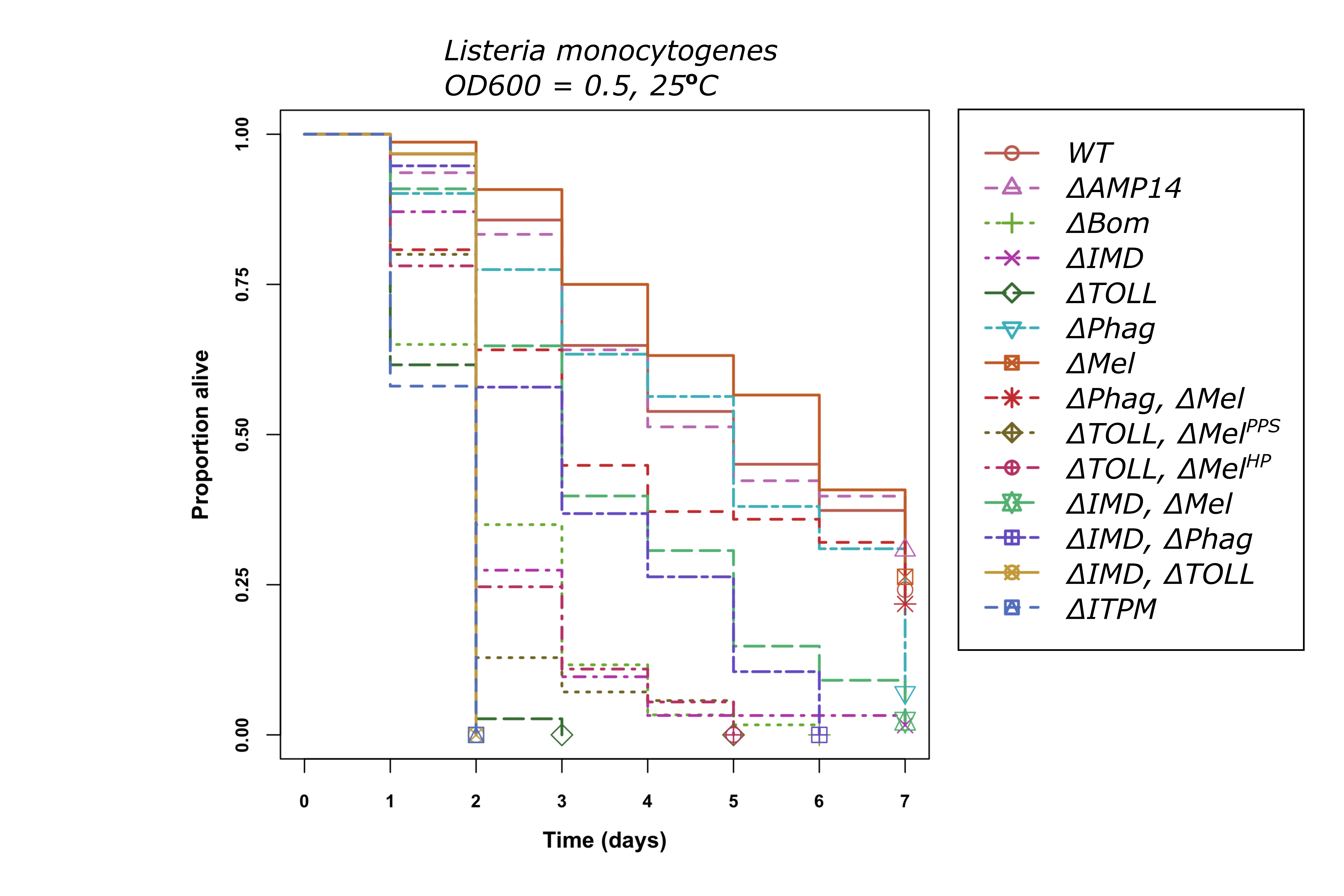

Supplement: Supplementary file 2. [file elife-107030-supp2.zip › Supplementary file 2/Gram-positive/L. monocytogenes.png]

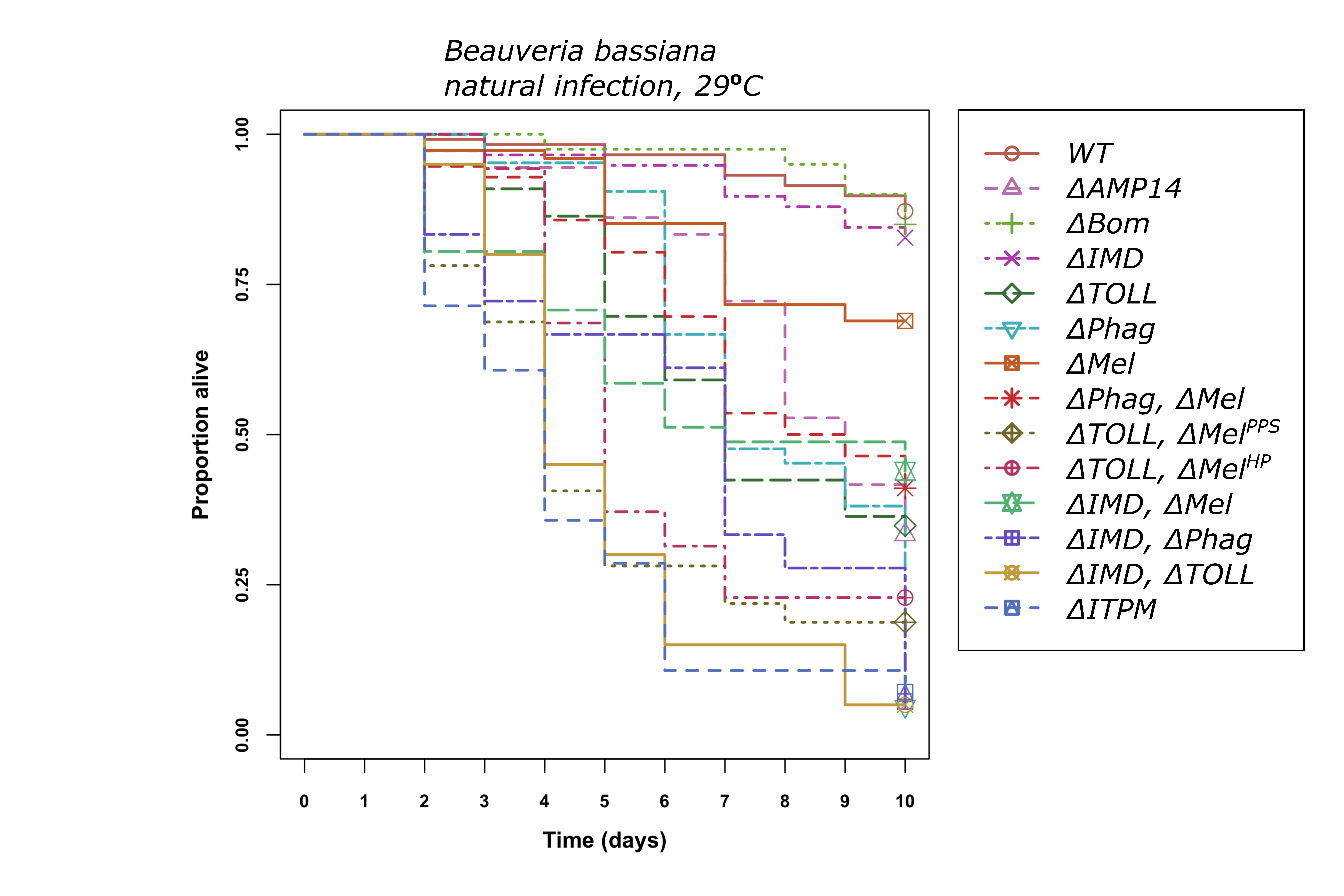

Supplement: Supplementary file 2. [file elife-107030-supp2.zip › Supplementary file 2/Fungi/B. bassiana NI.png]

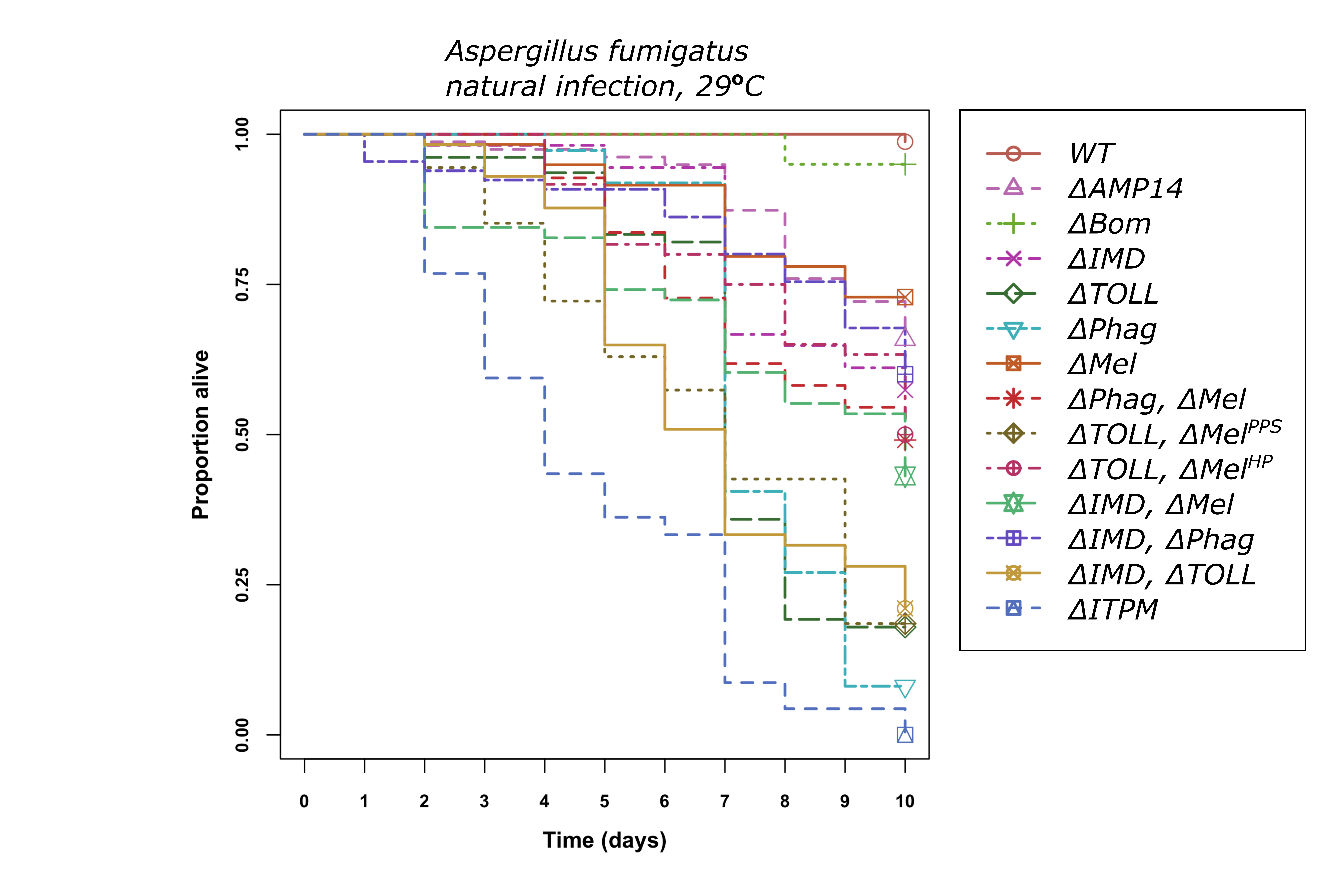

Supplement: Supplementary file 2. [file elife-107030-supp2.zip › Supplementary file 2/Fungi/A. fumigatus NI.png]

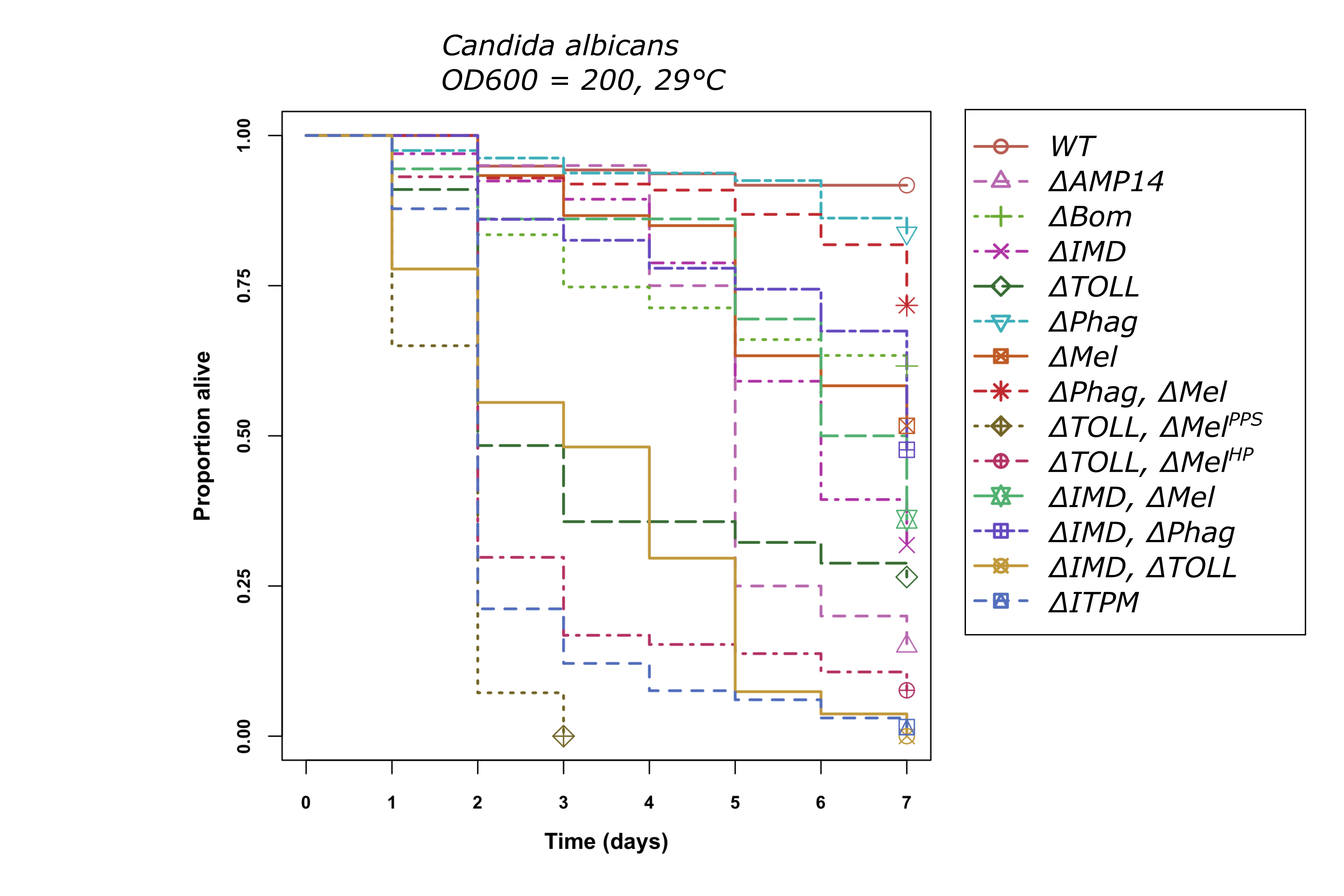

Supplement: Supplementary file 2. [file elife-107030-supp2.zip › Supplementary file 2/Fungi/C. albicans SI.png]

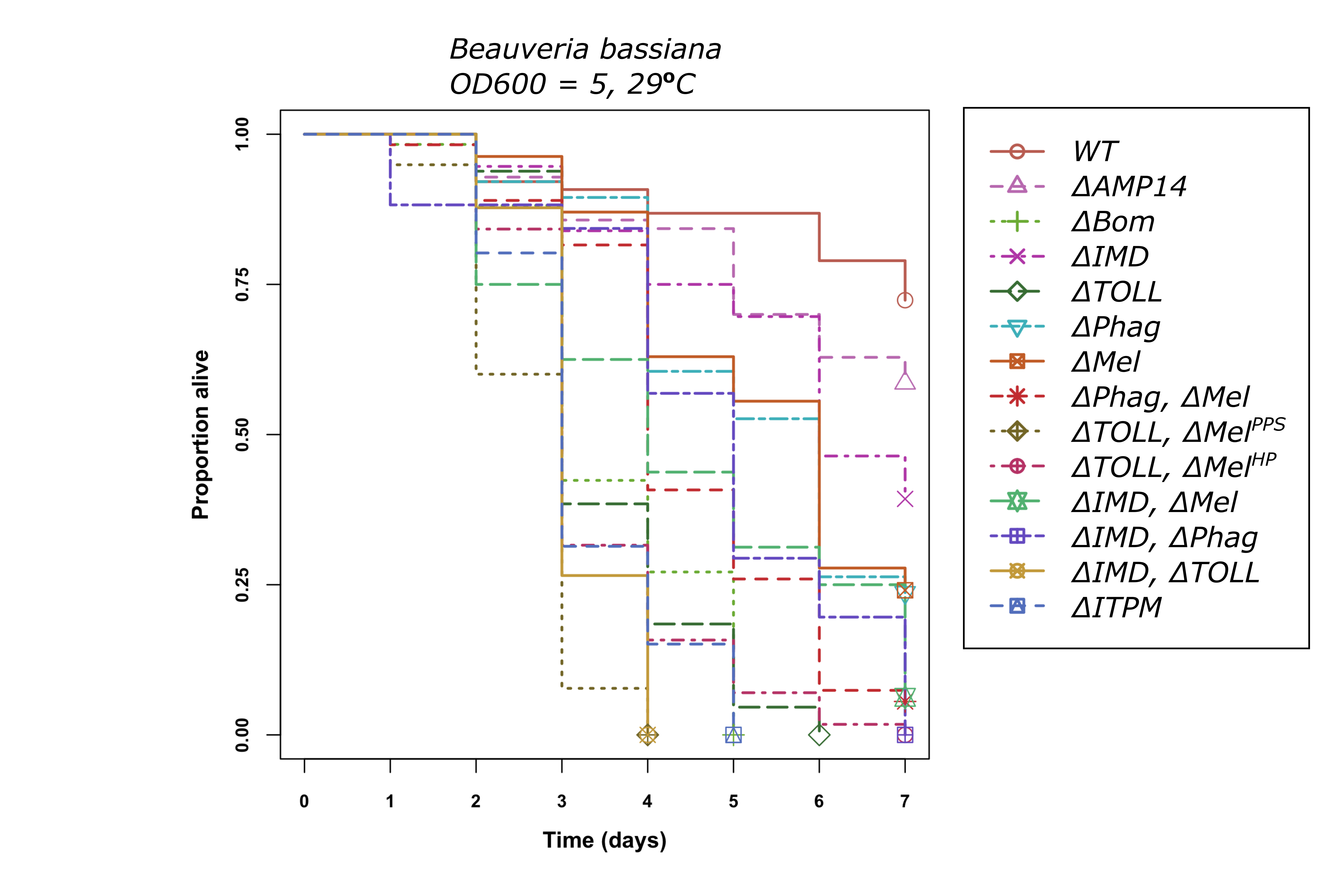

Supplement: Supplementary file 2. [file elife-107030-supp2.zip › Supplementary file 2/Fungi/B. bassiana SI.png]

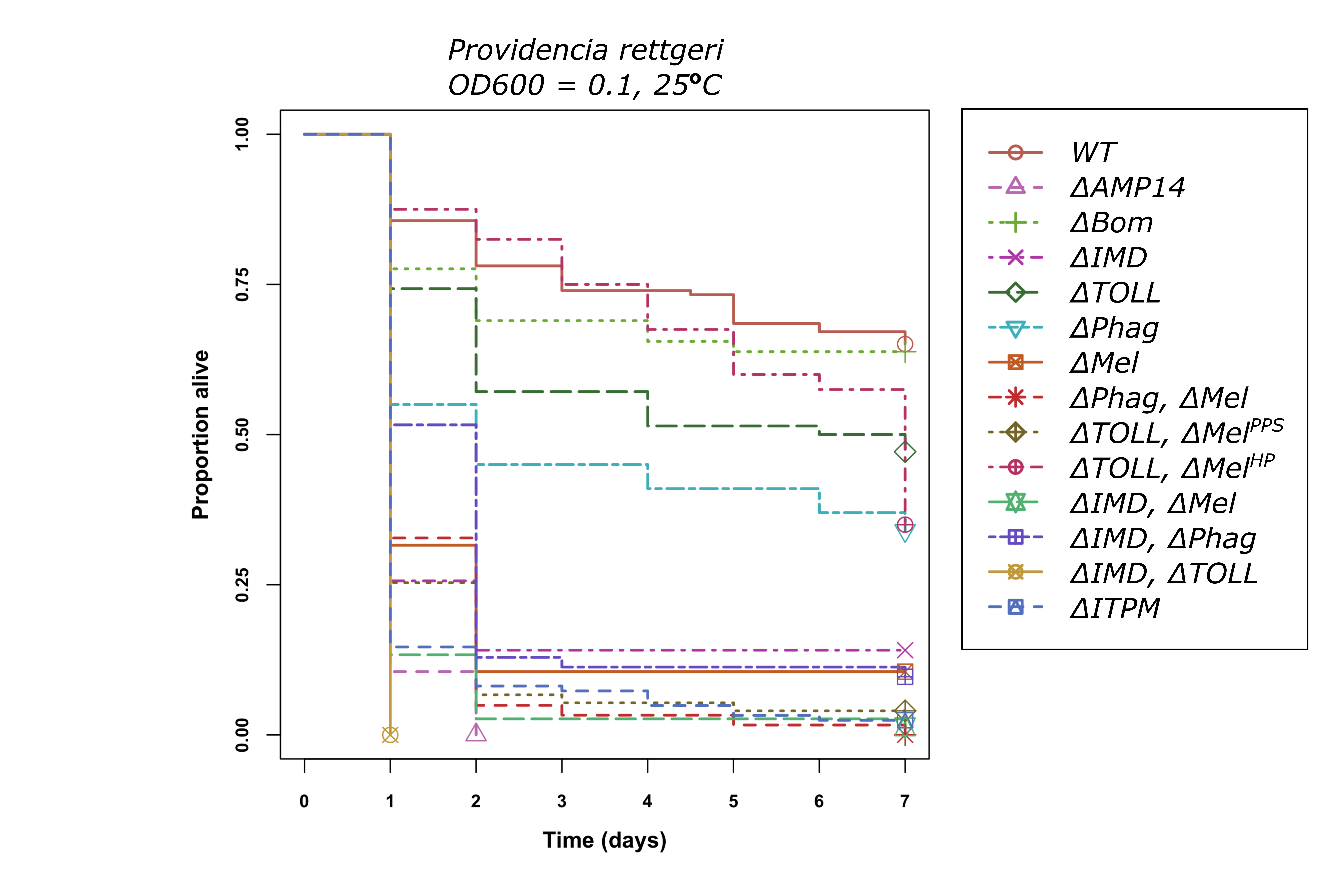

Supplement: Supplementary file 2. [file elife-107030-supp2.zip › Supplementary file 2/Gram-negative/Pr. rettgeri.png]

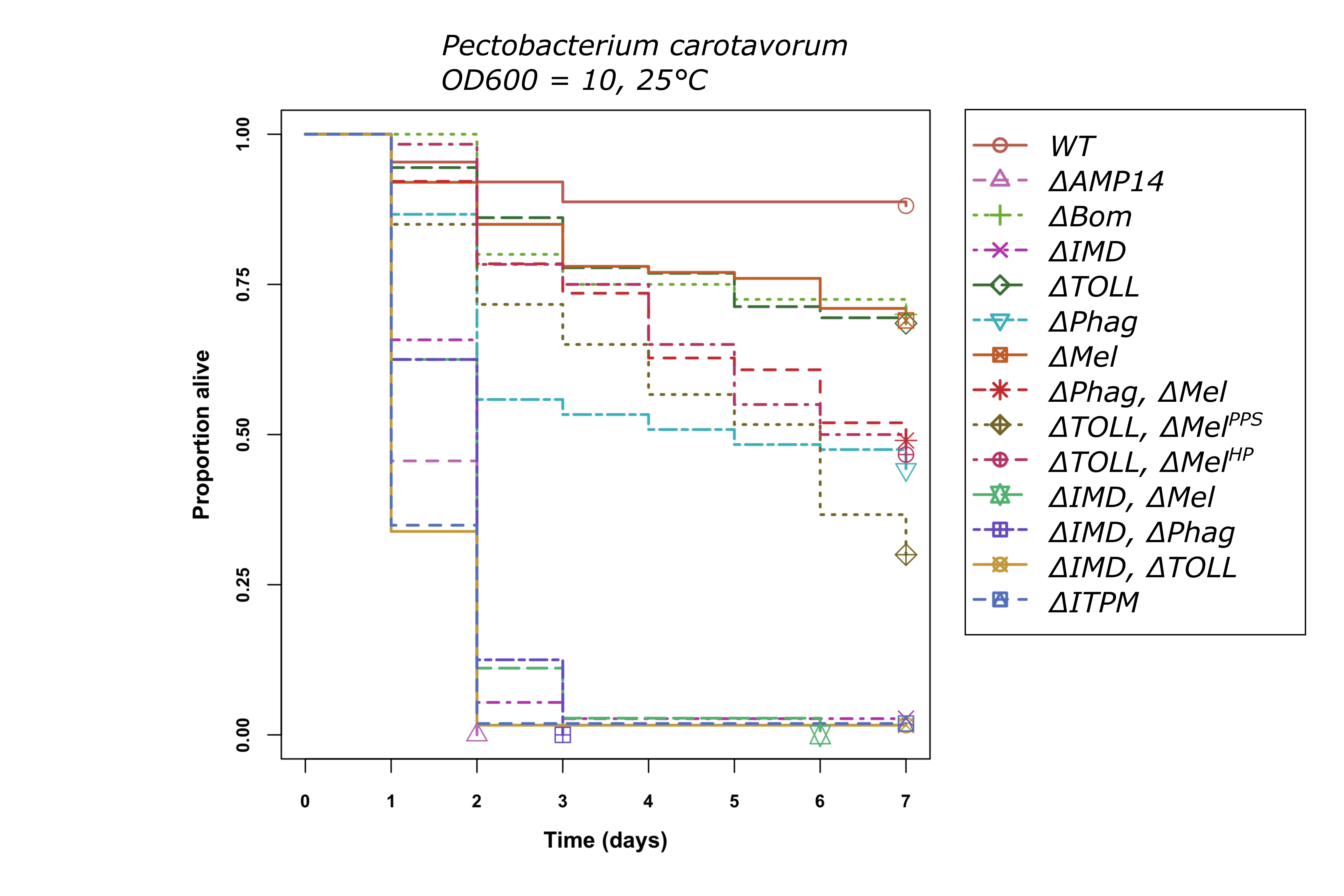

Supplement: Supplementary file 2. [file elife-107030-supp2.zip › Supplementary file 2/Gram-negative/Pe. carotovorum Ecc15.png]

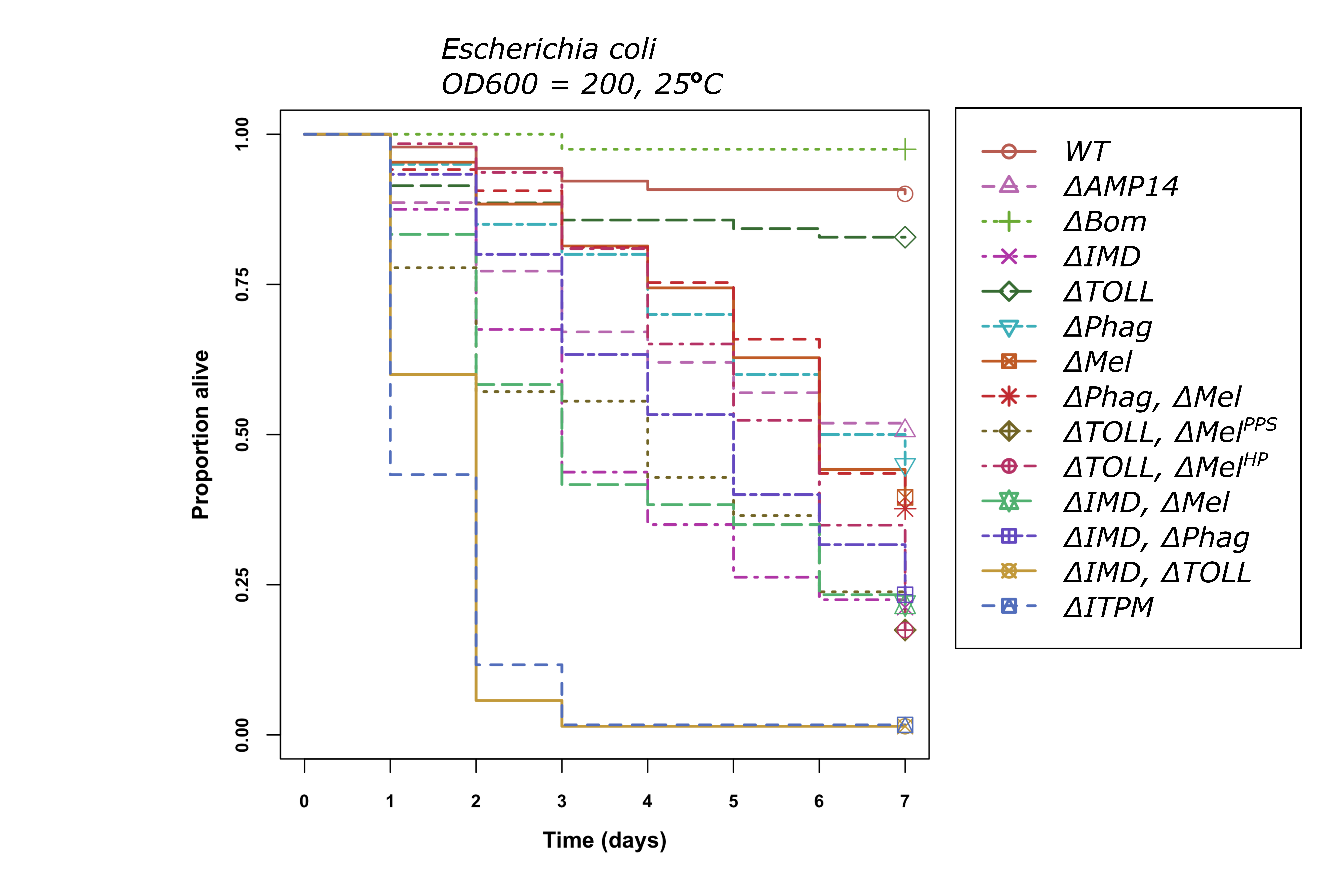

Supplement: Supplementary file 2. [file elife-107030-supp2.zip › Supplementary file 2/Gram-negative/E. coli.png]

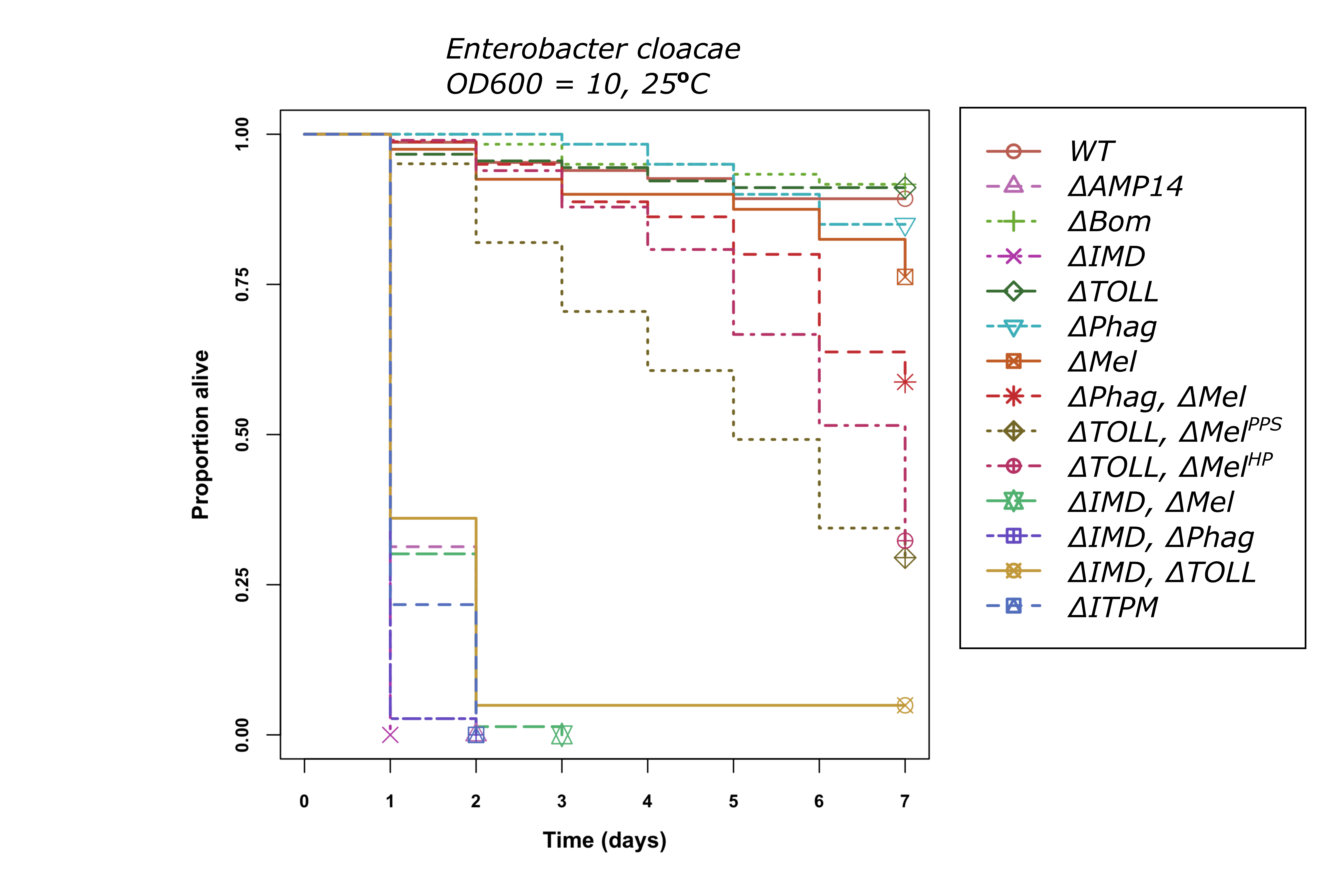

Supplement: Supplementary file 2. [file elife-107030-supp2.zip › Supplementary file 2/Gram-negative/E. cloacae.png]

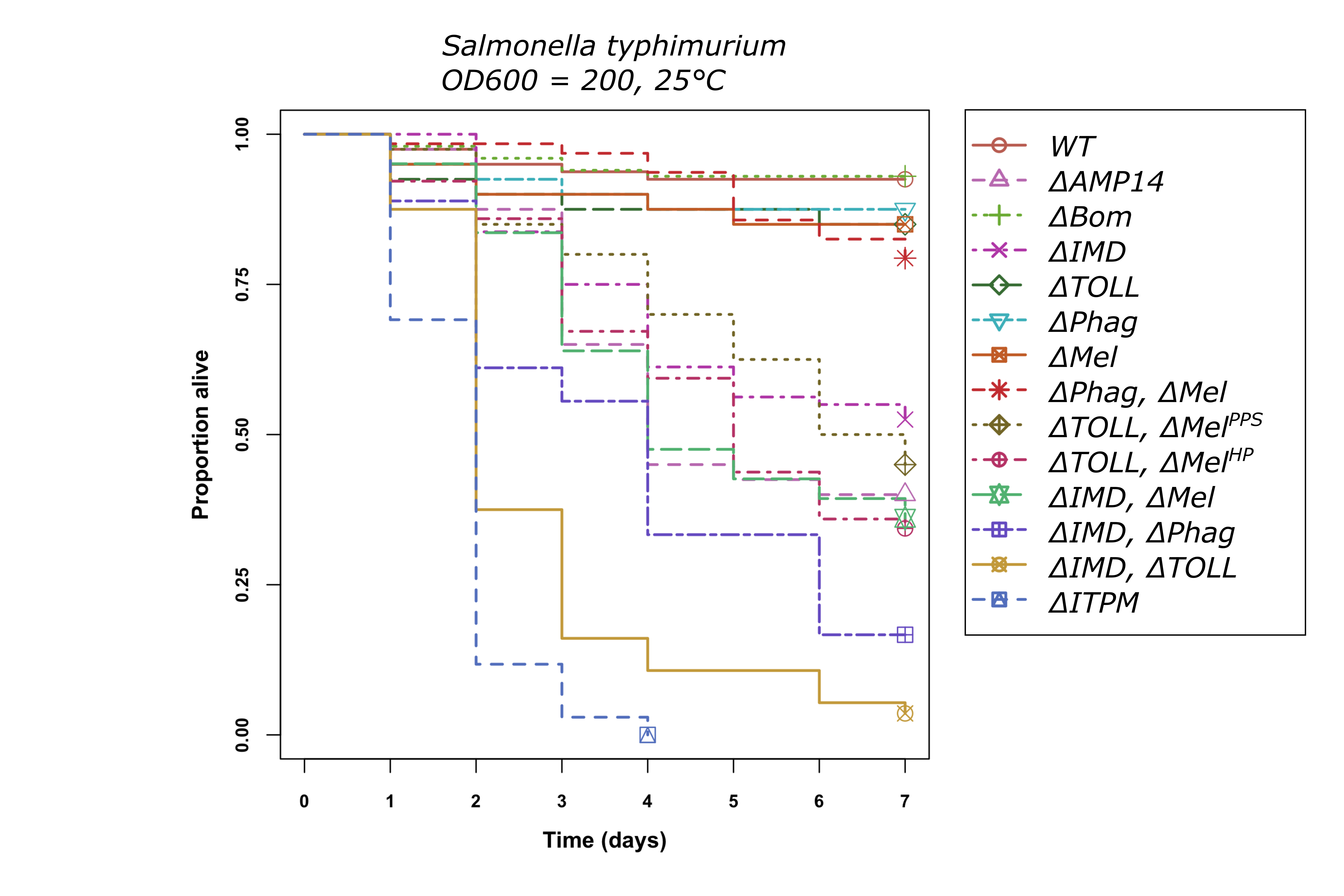

Supplement: Supplementary file 2. [file elife-107030-supp2.zip › Supplementary file 2/Gram-negative/Sa. typhimurium.png]

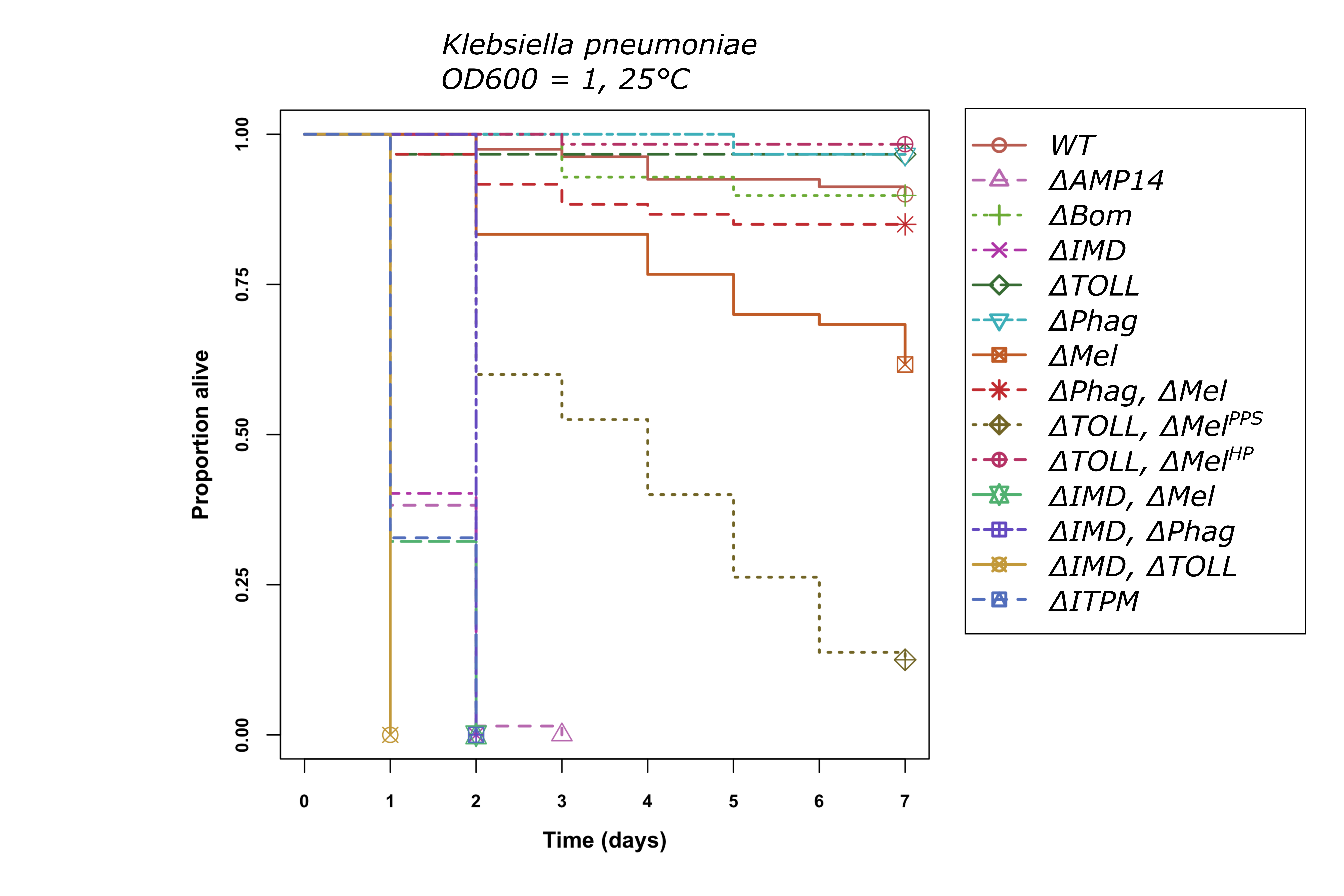

Supplement: Supplementary file 2. [file elife-107030-supp2.zip › Supplementary file 2/Gram-negative/K. pneumoniae.png]

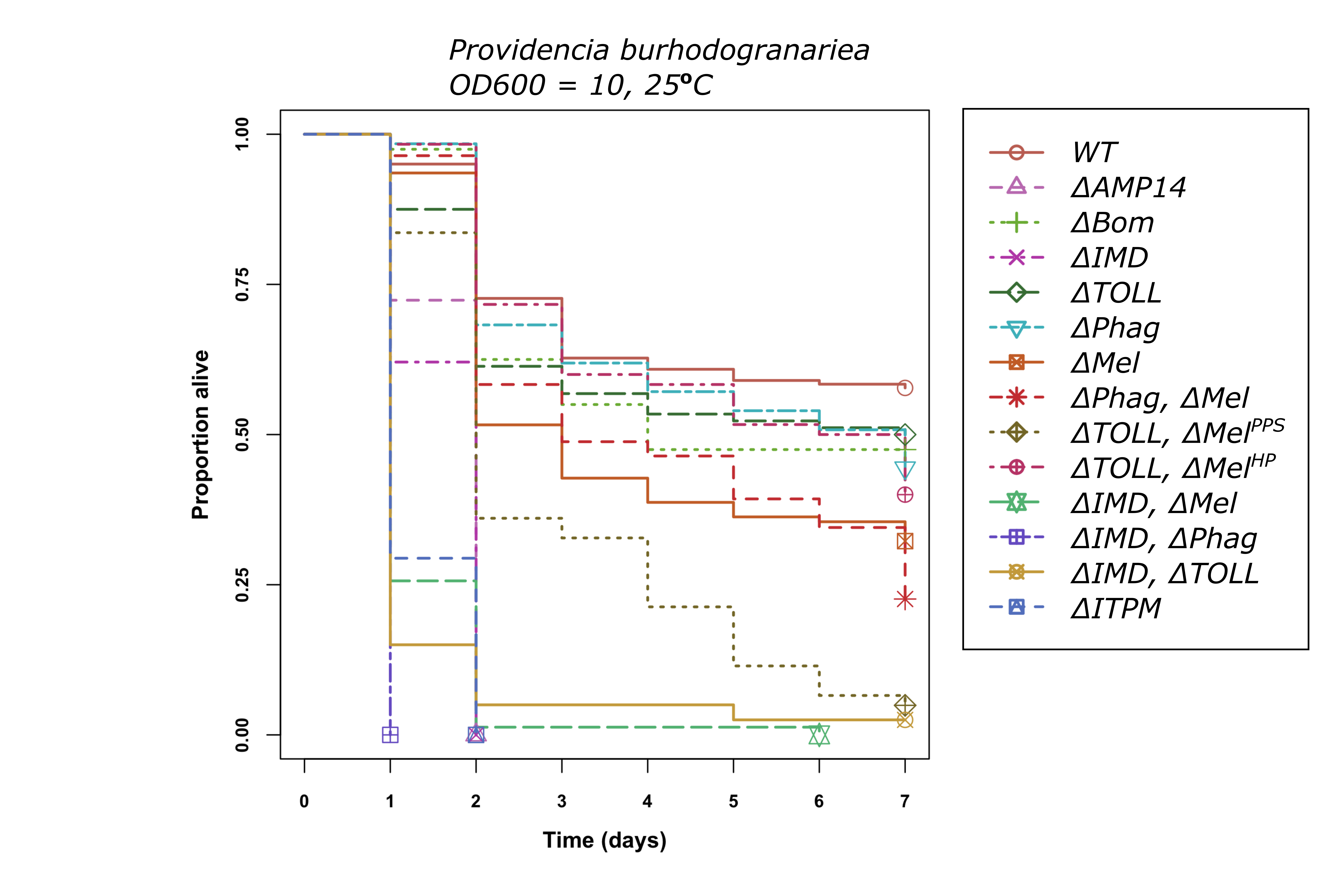

Supplement: Supplementary file 2. [file elife-107030-supp2.zip › Supplementary file 2/Gram-negative/Pr. burhodogranariea.png]

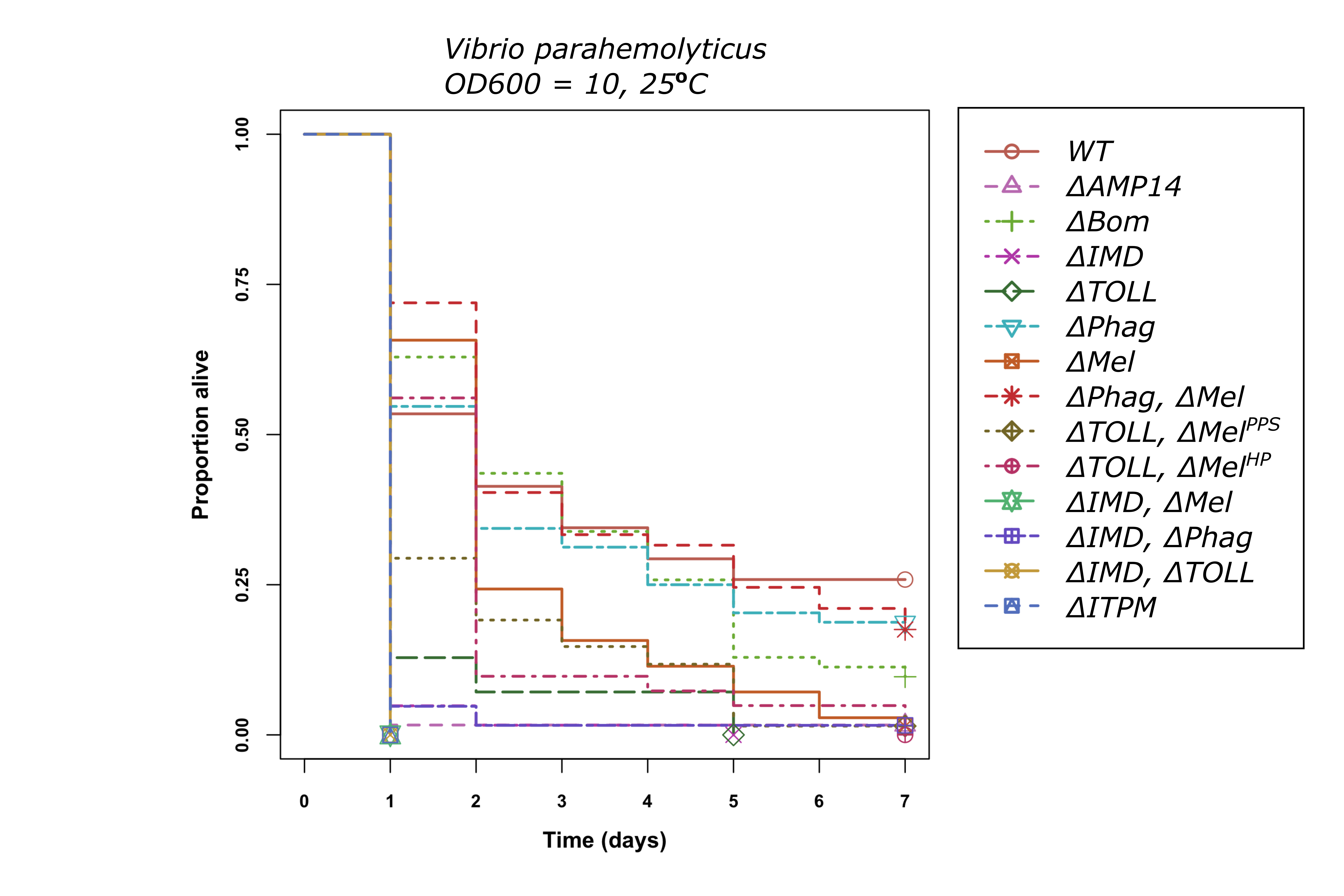

Supplement: Supplementary file 2. [file elife-107030-supp2.zip › Supplementary file 2/Gram-negative/V. parahemolyticus.png]

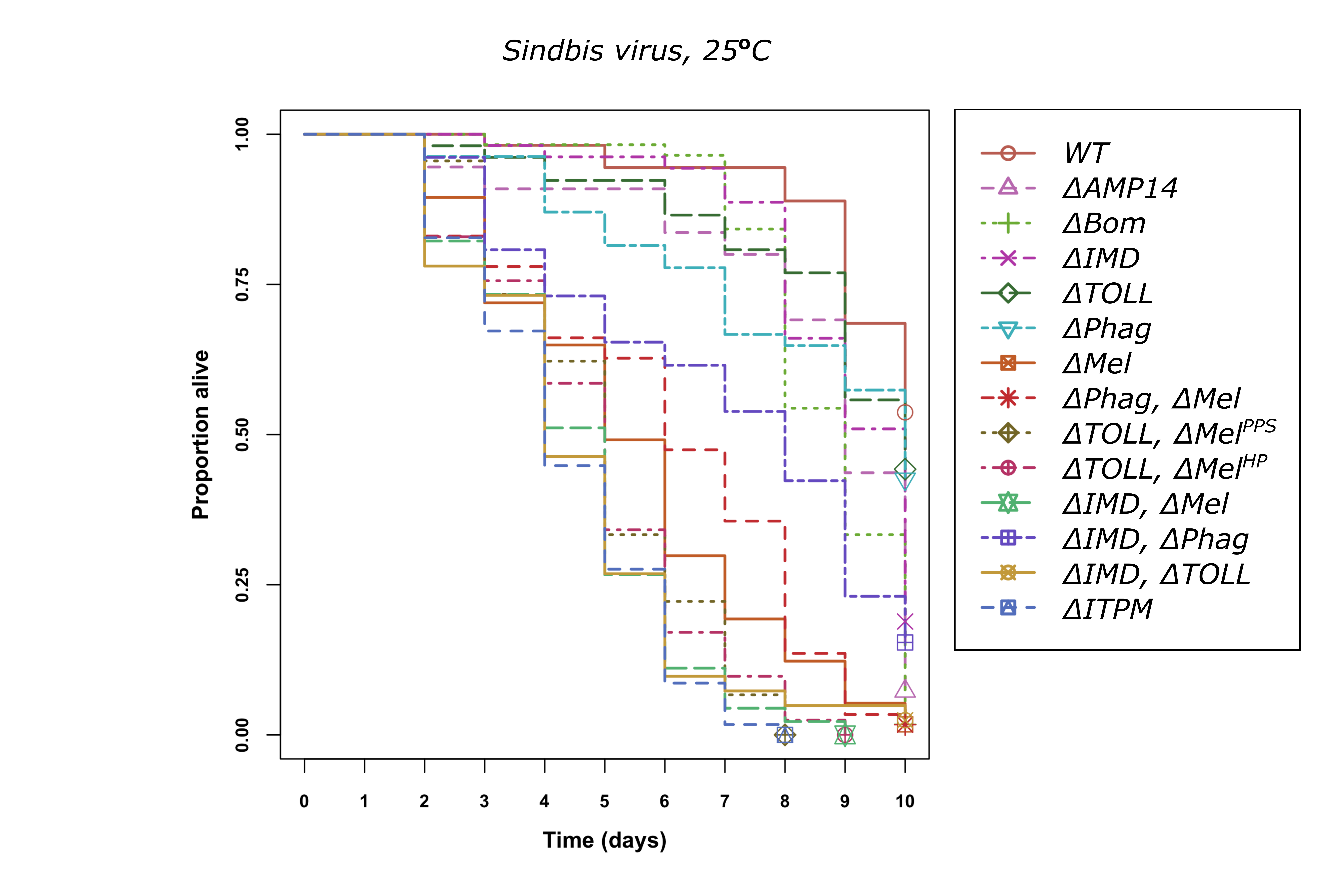

Supplement: Supplementary file 2. [file elife-107030-supp2.zip › Supplementary file 2/Virus/SINV.png]

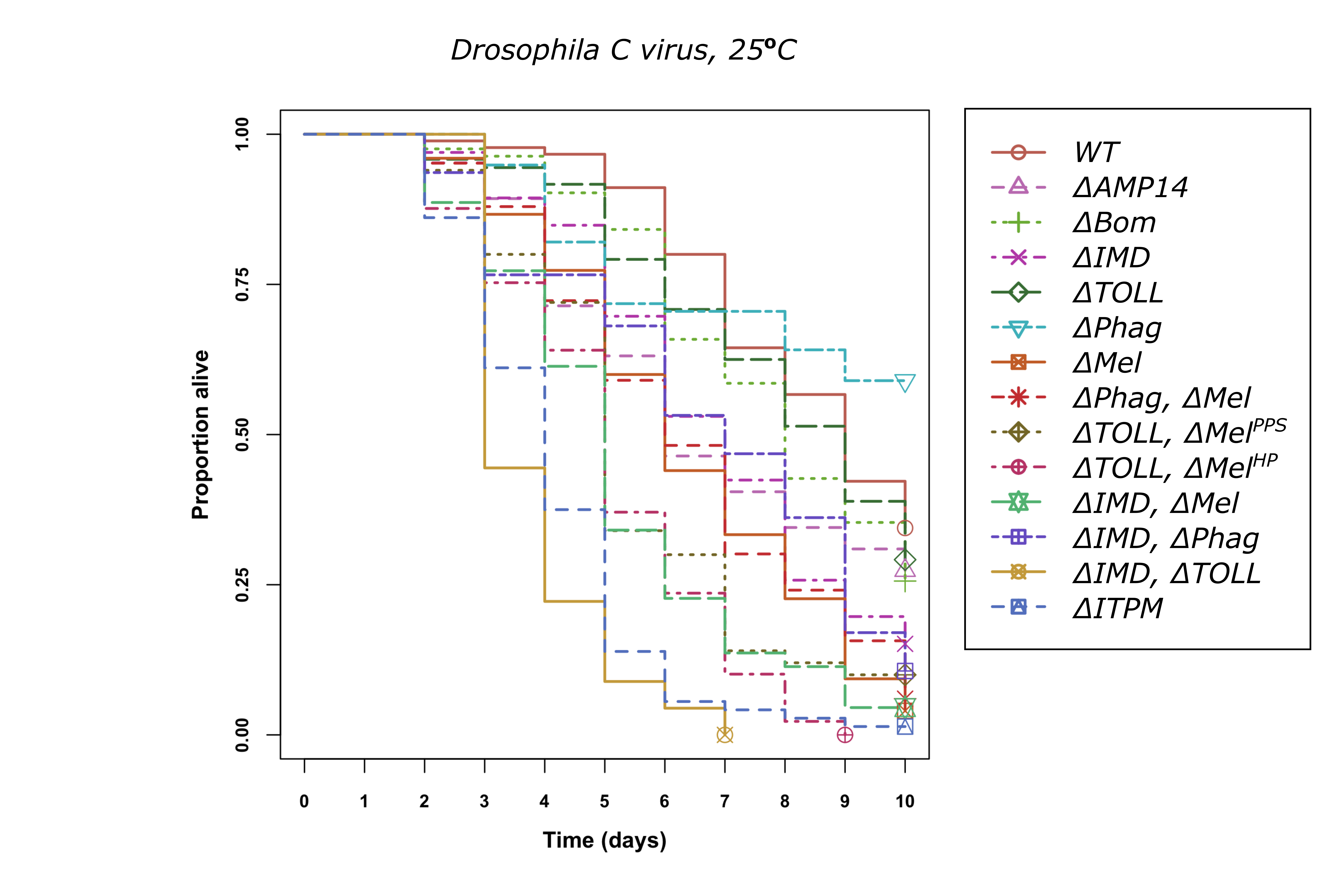

Supplement: Supplementary file 2. [file elife-107030-supp2.zip › Supplementary file 2/Virus/DCV.png]

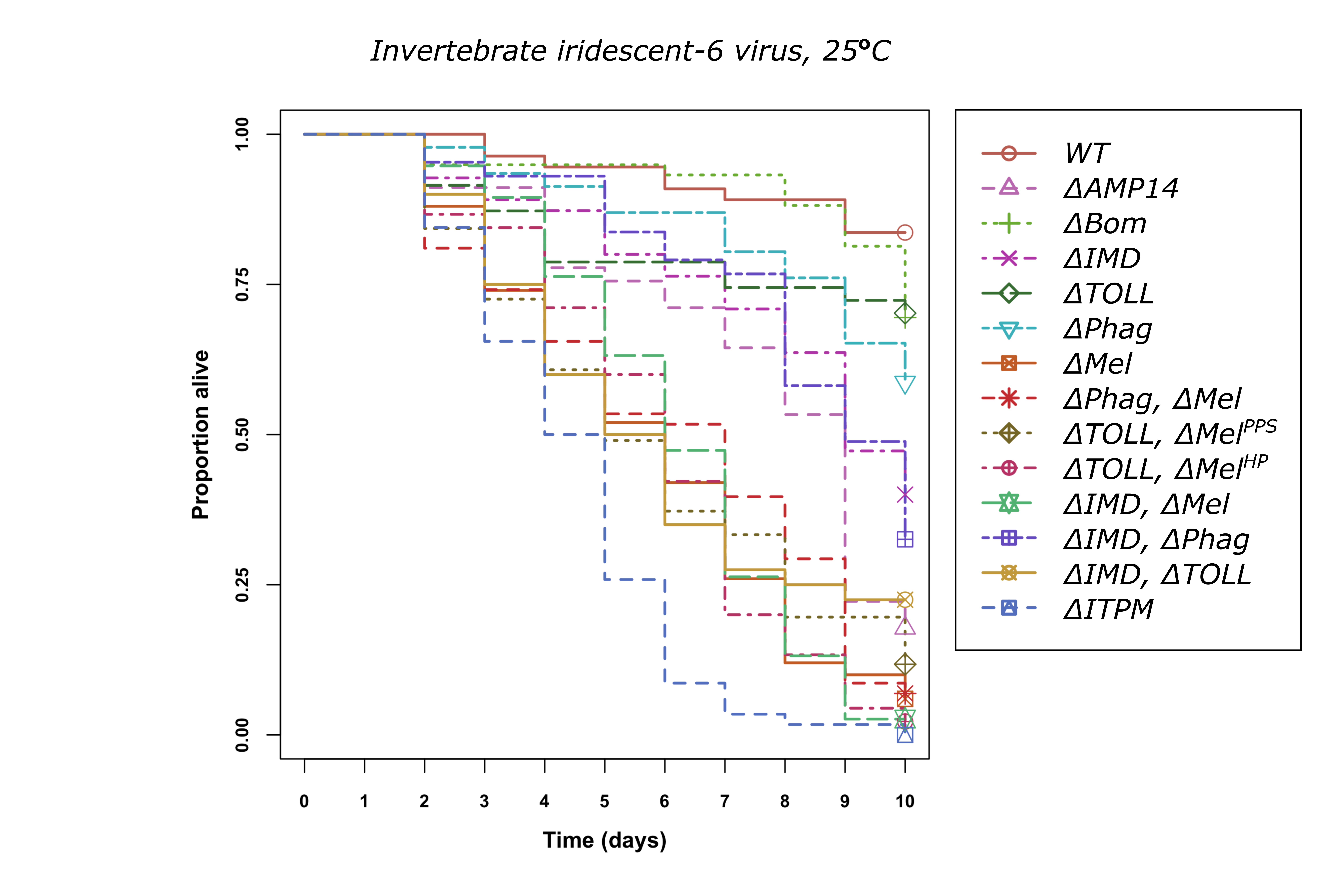

Supplement: Supplementary file 2. [file elife-107030-supp2.zip › Supplementary file 2/Virus/IIV6.png]

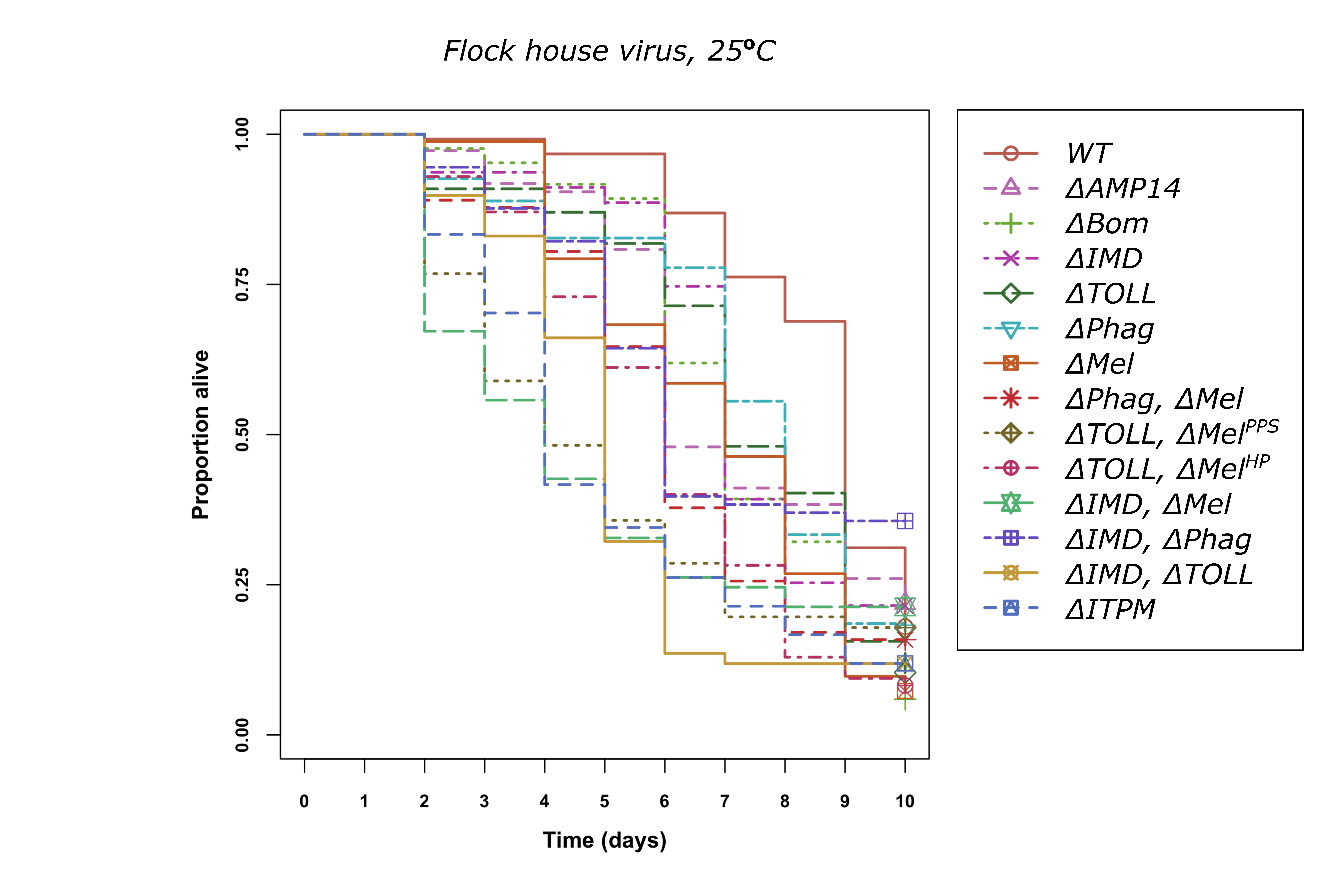

Supplement: Supplementary file 2. [file elife-107030-supp2.zip › Supplementary file 2/Virus/FHV.png]

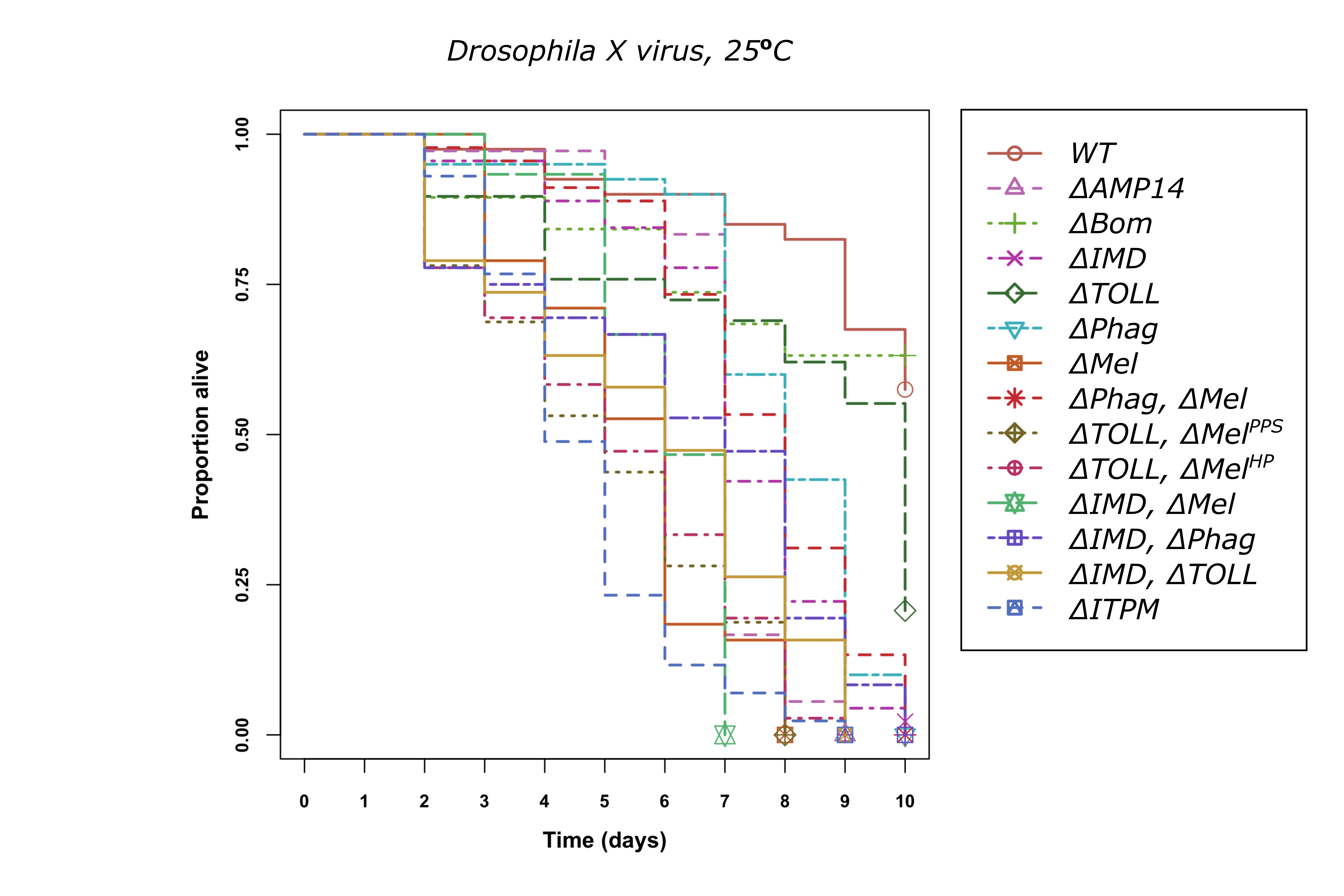

Supplement: Supplementary file 2. [file elife-107030-supp2.zip › Supplementary file 2/Virus/DXV.png]
